# Study of electric conduction mechanisms in bismuth silicate nanofibers

**DOI:** 10.1038/s41598-020-59563-6

**Published:** 2020-02-17

**Authors:** S. S. Batool, Z. Imran, Kamran Rasool, Jaweria Ambreen, Safia Hassan, Saira Arif, Mushtaq Ahmad, M. A. Rafiq

**Affiliations:** 1 Department of Physics, COMSATS University Islamabad, Park Road, Chak Shahzad Islamabad, 45550 Pakistan; 2 Micro and Nano Devices Group, Department of Metallurgy and Materials Engineering, Pakistan Institute of Engineering and Applied Sciences (PIEAS), P. O. Nilore, Islamabad, 4650 Pakistan; 3 Department of Chemistry, COMSATS University Islamabad, Park Road, Chak Shahzad 45550 Islamabad, Pakistan

**Keywords:** Electronic properties and materials, Electronic and spintronic devices

## Abstract

This work represents the nature of conduction mechanism in bismuth silicate (BiSiO) nanofibers as a function of temperature and frequency. Scanning electron micrographs and X-rays diffraction patterns exhibited the formation of cubic phases of Bi_4_(SiO_4_)_3_ and Bi_12_SiO_20_ nanofibers respectively with an average diameter of ~200 nm. Temperature dependent (300 K–400 K) electrical characterization of fibers was carried out in frequency range of ~20 Hz–2 MHz. The complex impedance analysis showed contribution from bulk and intergranular parts of nanofibers in conduction. Moreover, analysis of the Cole-Cole plot confirmed the space charge dependent behavior of BiSiO nanofibers. Two types of relaxation phenomena were observed through Modulus analysis. In ac conductivity curve, step like feature of plateau and dispersive regions were described by Maxwell-Wagner effect while the dc part obeyed the Arrhenius law. However, frequency dependent ac conductivity revealed the presence of conduction mechanism in diverse regions that was ascribed to large polaron tunneling model. Detailed analysis of complex Impedance and ac conductivity measurement showed negative temperature coefficient of resistance for the BiSiO nanofibers. Current-voltage (*IV*) characteristics represented ohmic conduction; followed by space charge limited current conduction at intermediate voltages. Results from both ac and dc measurements were in good agreement with each other.

## Introduction

Synthesis of bismuth silicate (BiSiO) nanostructures^1^ have gained importance due to its special applications in electro-optics, acousto-optics and piezoelectric industries^2^. High thermal stability, chemical durability, dielectric strength and interesting structure of BiSiO type materials are proved to be promising for electrical properties. This diversity of physical effects makes researchers to grow bismuth silicate from bulk to nanoscale. Different techniques have been adopted to synthesize bismuth silicate nanostructures such as solution phase synthesis^3^, hydrothermal, interfacial polymerization method and electrospinning^4,5^. Nanoscale materials have exclusive properties depending on their low dimensions and large surface area as compared to their bulk equivalents^6^. Among all reported nanostructured materials; one dimensional (1D) nanofibers are most common in electric device fabrications due to their large surface to volume ratio and relatively high crystallinity^7^. These 1D nanofibers are now frequently used in sensors, nano-electronics tissue engineering, photo-voltaic, nano-photocatalysts and nano-filtration^8^.The ac and dc conductivity of the bulk BiSiO materials have been reported^9^. The conductivity in these bulk materials is because of ionic conduction mechanism and governed by the mobility of bismuth (Bi) ions^10^. Effect of calcination on structural properties and consequently on the electrical properties for bulk BiSiO materials have been reported earlier^11,12^.

To report possible conduction mechanism along with dielectric properties of BiSiO type materials, scientists need to pay attention to the most probable causes of the change in its electrical behavior due to change in calcination temperature in ambient air. The main goal of the current work is to present an inclusive summary of electrical properties as ac and dc conductivity accompanied by electric modulus variation in BiSiO nanofibers as a function of frequency (1 Hz–2 MHz) and temperature (300 K–400 K). This type of comparison between ac and dc conduction transport on BiSiO nanofibers is rarely been found in literature.

## Materials and Methods

The BiSiO nanofibers used to study the electrical (ac and dc) properties were fabricated using electrospinning method.. Polyvinylpyrrolidone (PVP, M_w_ = 130k), Tetraethoxysilane (TEOS), N, N-Dimethylformamide (DMF), Bismuth Acetate (Bi(C_2_H_3_O_2_)_3_), Acetic acid (CH_3_COOH), and ethanol (C_2_H_5_OH) were obtained from Sigma Aldrich and used as precursor materials. Polymer solution was prepared by adding 1 g PVP/10 ml ethanol and 2 ml TEOS/1 ml acetic acid. The mixture was stirred for 15 minutes, followed by the gradual addition of 1.0 g of bismuth acetate [Bi(C_2_H_3_O_2_)_3_] dissolved in 1 ml of DMF. The whole solution was stirred for about 90 minutes in a capped beaker. The final solution was loaded into a syringe and a voltage of 10 kV was applied between the tip of the needle and collector plate^13,14^. As spun PVP/ bismuth silicate nanofibers were collected on a copper plate. The as prepared nanofibers were heat-treated at 600 °C for 6 h in air furnace.

**Figure 1:**
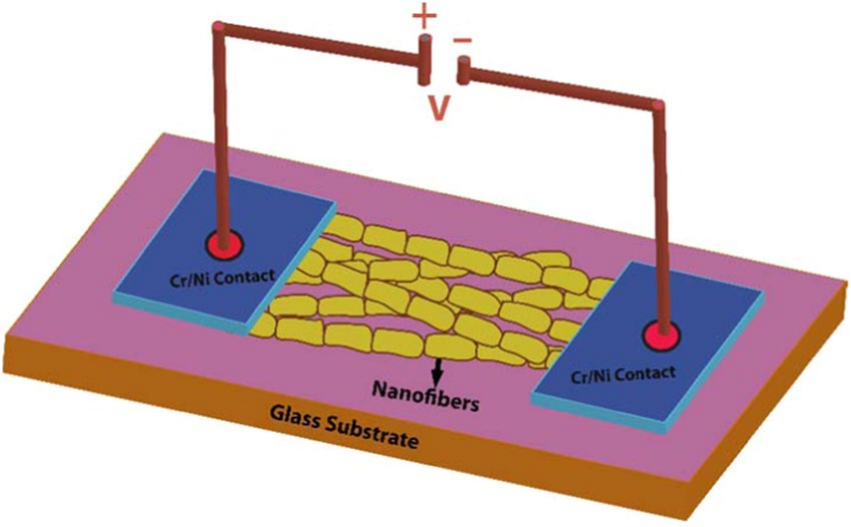
Schematic diagram of the final fabricated device.

The device of BiSiO was fabricated for the electrical properties measurements. For this purpose, 100 nm^2^ electrodes were made by depositing chromium (Cr) followed nickel (Ni) on a glass substrate to improve the adhesion of Ni to the glass substrate. The suspension of BiSiO nanofibers in isopropanol were prepared by using ultrasonic agitation bath. The nanofibers were then deposited on the device using drop casting method. Figure 1 shows the schematic diagram of the final fabricated device.

## Results and Discussions

**Figure 2:**
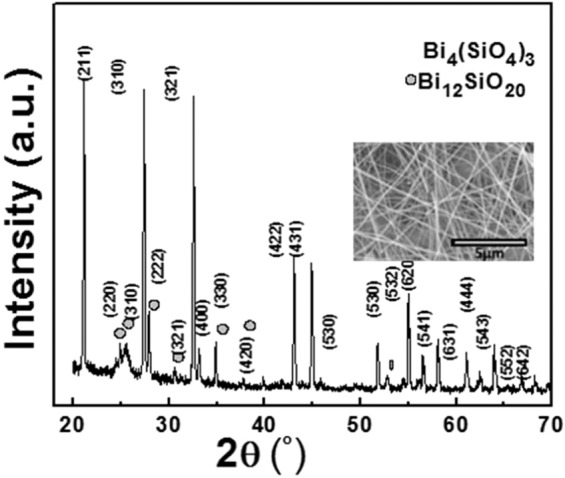
XRD pattern of Bismuth Silicate nanofibers and Inset shows SEM image of Bismuth Silicate nanofibers.

The morphological studies of the BiSO nanofibers were carried out using Scanning electron micrographs (SEM) and X-ray diffraction Analysis (XRD) as shown in Fig. 2. SEM image (inset Fig. 2) revealed the formation of nanofibers with diameter of less than 500 nm and length of several micrometers. XRD patterns confirmed the formation of cubic Bi_4_(SiO_4_)_3_ nanofibers. Maximum peaks were indexed with JCPDS card number 01-080-1596^15^, whereas, few peaks were indexed with the JCPDS card number 00-037-0485 and are attributed to Bi_12_SiO_20_ phase. This may be due to high temperature heat treatment *i.e*. 600 °C. Texture coefficient (*T*_*c(hkl)*_) of the sample was calculated to check the random or preferential growth of the Bi_4_(SiO_4_)_3_ phase using the formula 1 given below^16^. The intensity of peaks related to Bi_12_SiO_20_ phase is very low. Therefore, it is very difficult to estimate the preferential growth direction.

1 $${T}_{c(hkl)}=\frac{{I}_{hkl}/{I}_{r(hkl)}}{1/n{\sum }_{n}{I}_{hkl}/{I}_{r(hkl)}}$$

*I*_*hkl*_ shows the intensities in XRD spectra of nanofiber mats and *n* is the number of diffraction peaks selected for the calculation of *T*_*c(hkl)*_. I*r(hkl)* is the intensity of the reference pattern (JCPDs card). The deviation from unity or higher values of *T*_*c(hkl)*_ shows the preferred orientation. Growth in preferred orientation along the particular plane shows the maximum number of grains along that plane^15^.It is reported that an increase in texture co-efficient from 1 shows a higher degree of preferred orientation along the particular plane. Moreover, the higher or lower values of texture co-efficient from unity relates the increase or decrease in planar density beside a specific crystal plane^16^. The texture analysis of BiSiO nanofibers shows that the fibers are highly textured along (321) and (532) plane with planar density as shown in Table 1.

**Table 1:** Calculated Texture co-efficient calculation for different planes in XRD spectrum.

h	k	l	I_xrd_	I_card_	Texture coefficient
2	1	1	100	100	1.06
**3**	**1**	**0**	**98**	**90.7**	**1.14**
3	2	1	99	86.9	1.20
4	0	0	14	12.9	1.14
4	2	2	42	43.5	1.02
4	3	1	39	42.1	0.98
5	3	0	15	14.7	1.07
5	3	2	33	29.2	1.19
6	2	0	9	10.3	0.92
5	4	1	17	15.9	1.13
6	3	1	13	12.2	1.12
5	4	3	15	15.4	1.03

**Figure 3:**
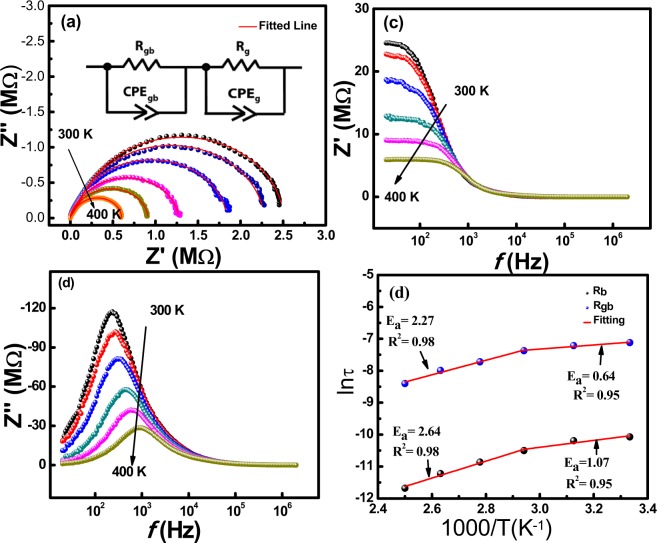
Impedance analysis of Bismuth Silicate nanofibers **(a)** Cole-Cole Plot **(b)** Real part of Impedance with frequency **(c)** Imaginary Part with frequency curves at different Temperatures **(d)** Relaxation time with absolute temperature.

Impedance analysis were done at a temperature range (*i.e*. 300 K–400 K) for BiSiO nanofibers and results are illustrated in Fig. 3(a–c). Figure 3(a) displays the complex impedance plane plot (*Z*″ *vs. Z*′) which provides data in the form of arcs with relaxation time (τ = RC, where τ, R and C are the relaxation time, resistance and capacitance of the charge carriers, respectively). Arrows point towards increasing temperature. The *Z*″ *vs. Z*′ arcs for all particular temperatures have depressed semicircles indicating that there are multiple relaxations present in our material. These multiple relaxations are confirmed by fitting the data with Z-View software with fitting error <5%. Scattered points in the Fig. 3(a) are the experimental data; whereas the red line shows the fitted line. The combination of two parallel RC circuits are shown in inset of Fig. 3(a). The values of fitted parameters: bulk resistance and grain boundary resistance (*R*_*b*_ and *R*_*gb*_), pseudocapacitance (*C*_*b*_ and *C*_*gb*_)and *n* parameters *n*_*b*_ and *n*_*gb*_ are shown in Table 2. This equivalent circuit signifies the two different routes for conduction mechanism in BiSiO nanofibers. The different nature of arcs at different temperatures and frequencies provides various signs of materials. The presence of two semicircles are indicating electrical contribution from grain interior (bulk) and due to intergranular activities (grain boundaries effects). Following relations are used to represent the real (*Z*′) and imaginary (*Z*″) part of impedance^17^:

2 $${\rm{Z}}{\prime} =\frac{{R}_{b}}{[1+{(\omega {R}_{b}{C}_{b})}^{2}]}+\frac{{R}_{gb}}{[1+{(\omega {R}_{gb}{C}_{gb})}^{2}]}$$

and

3 $${{\rm{Z}}}^{{\prime\prime} }=\frac{\omega {{R}_{b}}^{2}{C}_{b}}{[1+{(\omega {R}_{b}{C}_{b})}^{2}]}+\frac{\omega {{R}_{gb}}^{2}{C}_{gb}}{[1+{(\omega {R}_{gb}{C}_{gb})}^{2}]}$$

Where ω is the angular frequency. The electrical behavior of semiconducting BiSiO nanofibers can be interpreted by these semicircle curves and their point of intercepts on the real (*Z*′) axis. These intercepts give the values of the bulk (*R*_*g*_) and grain boundaries (*R*_*gb*_) influences in resistance. The maxima of arcs provide the frequency *ω*_*max*_ for each *RC* element and is represented by ${\omega }_{max}=2\pi {f}_{max}=\frac{1}{RC}=\frac{1}{\tau }$ whereas the time constant ‘*τ*’ and ‘*f*_*max*_’ are independent of sample geometry and are the intrinsic properties of *RC* element^18^. Bulk capacitance is calculated by the formula.

**Table 2:** *RC* circuit fitting parameters (error <5%) of complex impedance semicircles at different temperatures (300 K–400 K).

Temp. (K)	R_g_ (MΩ)	CPE_g_	n_g_	C_g_ (F)	R_gb_ (MΩ)	CPE_gb_	n_gb_	C_gb_ (F)
300	2.38	3.72 × 10^−10^	0.96	1.91 × 10^−10^	0.13	4.04 × 10^−10^	0.98	3.44 × 10^−10^
320	2.22	4.30 × 10^−10^	0.94	2.77 × 10^−10^	0.08	4.41 × 10^−10^	0.97	3.21 × 10^−10^
340	1.78	4.10 × 10^−10^	0.95	2.81 × 10^−10^	0.07	3.58 × 10^−10^	0.96	2.31 × 10^−10^
360	1.19	3.75 × 10^−10^	0.97	2.94 × 10^−10^	0.06	6.54 × 10^−10^	0.94	3.45 × 10^−10^
380	0.84	3.30 × 10^−10^	0.99	3.05 × 10^−11^	0.05	8.85 × 10^−10^	0.90	2.93 × 10^−10^
400	0.56	2.94 × 10^−10^	1.06	5.15 × 10^−10^	0.04	8.17 × 10^−10^	0.91	1.05 × 10^−10^

$C={(CPE)}^{1/n}\ast {R}^{(1-n)/n}$, where *CPE* is the constant phase element and ‘*n*’ is empirical constant. The value for *n* represents the deviation from ideal Debye behavior and the reported value is one for ideal capacitor and zero for ideal resistor^19^. Resistance and capacitance values of bulk and grain boundaries are obtained after equivalent *RC* circuit fitting of complex impedance semicircles at different temperatures (Table 2). It is obvious that the values of *R*_*b*_ and *R*_*gb*_ decrease with increase in temperature; which confirms the negative temperature coefficient of resistivity (NTCR) behavior same as present in semiconductors^20^.

A detailed analysis of the frequency dependent *Z*′ and *Z*″ was carried out to understand the crossover from resistive to conductive behavior less than the cut-off frequency *f*_*o*_. Figure 3(b) represents the real impedance curve (*Z*′) as a function of frequency at different temperatures. At low frequency region, a decrease in *Z*′ values is observed with temperature. This behavior represents the existence of a negative temperature coefficient of resistance (NTCR) in BiSiO nanofibers^21–23^. It is also observed that all spectrums merged into a single spectra at relatively high frequencies (0.01 MHz) independent of temperature change, which exhibited clear indication of space charge dependent behavior of the BiSiO nanofibers^24^. Essentially, the charges accumulated near boundaries have enough energy to overcome the barrier; consequently leading to an improvement in conductivity. The active distance between junctions in BiSiO nanofibers has defined the specific length of the conductive network. This length is probed when it is equivalent to ac modulation period, therefore allowing the transportation of charges from one junction to another along the BiSiO nanofibers. Below *f*_0_, the charge carriers penetrate at greater distances andare analogous to the scale of whole nanofibers network in the low frequency region. In this range, the impedance is formally corresponding to the *dc* resistance. For frequencies greater than *f*_0_, the carriers permeate at a smaller distance into each *ac* phase with charge carriers only inclining to travel within nanofibers. The changeover from extended range transport to localized carrier confinement is described by *f*_0_ in *Z*′. The term *f*_0_ is also associated with maximum peaks in *Z*″ (Fig. 3(c)). The trend displays, *f*_0_ fluctuating to higher frequencies with temperature and *Z*′ consistently decreases. This corresponds to the higher level of connectivity between fibers and clear crossover to conducting behavior in addition to variations in the effective lengths between junctions^24^.

Figure 3(d) represents the effect of temperature on relaxation time (*τ*) calculated from the relaxation frequency that is obtained from fitting results of the complex impedance plots as a function of frequency. This plot follows the Arrhenius relation as^25^:

4 $$\tau ={\tau }_{o}\exp [\frac{-{E}_{a}}{{k}_{B}T}]$$

Where, *τ*_*o*_ is the pre-exponential factor called characteristic relaxation time, *T* is the absolute temperature, *E*_*a*_ is the activation energy of the relaxation process, estimated from the slope of *ln τ vs. 1000/T*, and *k*_*B*_ is the Boltzmann constant. *E*_*a*_ values for the BiSiO nanofibers are found to be 2.64 eV and 1.07 eV for bulk part and 2.27 eV and 0.64 eV for intergranular part respectively. *E*_*a*_ values are found to be elevated at low temperatures and decreased at relatively higher temperatures region for both bulk and intergranular interferences.

**Figure 4:**
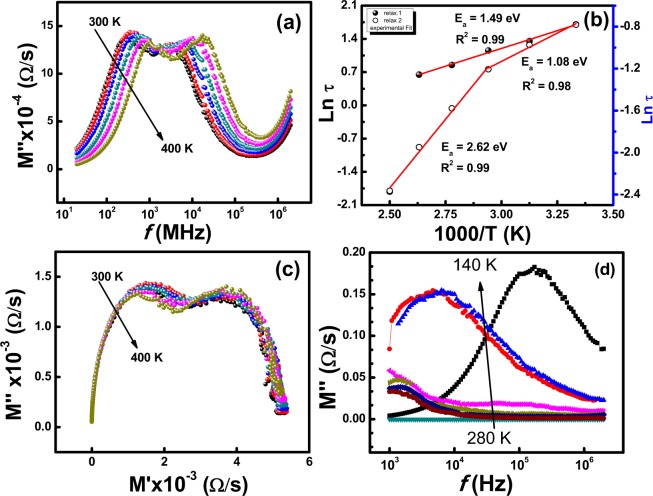
(**a**) Effect of frequency and temperature on the imaginary part of the electric modulus (*M*′′) (**b**) Activation energy calculation (**c**) complex plane plot of *M*′′ vs. *M*′ at different temperatures and (**d**) imaginary part of the electric modulus (*M*′′) at low temperature (280 K- 140 K).

**Figure 5:**
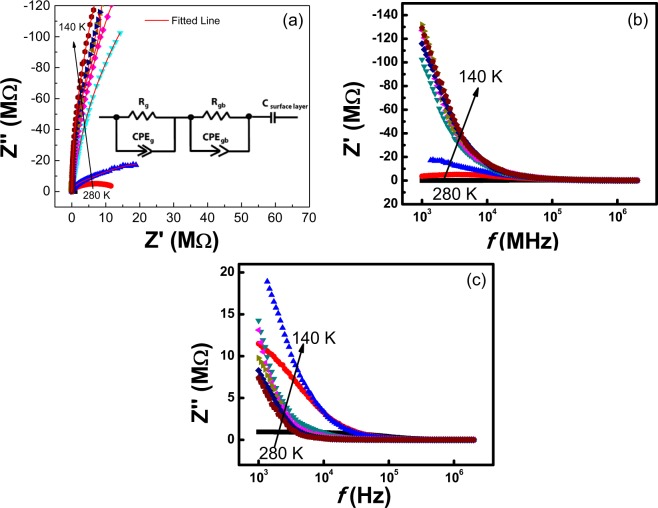
Impedance analysis of Bismuth Silicate nanofibers in the temperature range of 280 K–140 K (**a**) Cole-Cole Plot. (**b**) Real part of Impedance with frequency **(c)** Imaginary Part with frequency curves at different Temperatures.

The modulus analysis is performed for detailed analysis of space charge relaxation phenomenon observed in complex impedance formulism. This approach also shows the space charge phenomena through the least capacitance and it can overcome the impact of electrode polarization^26^. Figure 4(a) depicts the effect of frequency and temperature (300 K–400 K) on the imaginary part of the electric modulus (*M*′′). We can see two dielectric relaxations. The activation energies (*E*_*a*_) for two relaxation processes corresponding to grain and intergranular are calculated using Arrhenius equation (Eq. 4) and found to be 1.49 eV and 2.62 eV respectively (Fig. 4(b)). Figure 4(c) represents the complex plane plot of *M*′′ vs. *M*′ at higher temperatures that also shows two dielectric relaxations. A close inspection of the *M*′′ spectroscopic plot shows the presence of a peak at high frequency, whose maximum is above 100 MHz as the *M*′′ data do not tend to be zero at high frequency; it indicates that the sample possibly contains a third component which coulds probably be associated with the bulk response. In order to see this phenomenon, *M*′′ variations against frequency at lower temperatures 140 K–280 K are done and shown in Fig. 4(d). Furthermore, fitting of RC circuits to the impedance data collected at below room temperature shows that there is an additional part contributing to the impedance. The additional part is found by placing a parallel capacitance component with other two RC circuits as shown in Fig. 5(a). This is observed at all temperatures below 300 K. The capacitance values are added to the Table 3. The calculated capacitance values lie in the range 10^−7^ to 10^−9^ F, therefore this effect is attributed to the surface layer effect. As we increase the temperature from 280 K to the room temperature and above, this additional part vanishes. The fitting with additional capacitance component gives error and it works with only two RC circuits as explained earlier. Therefore, we conclude that below room temperature there are three regions present whereas there are two regions from 300 K to 400 K. The reason of vanishing this additional component at higher temperatures may be due to non-active charge carriers near surface at low temperatures. As we increase the temperature these charge carriers get energy to contribute to conduction and relative capacitive part vanishes. Figure 5(a–c) shows the impedance data collected below room temperature. The fitted parameters at low temperature are shown in Table 3.

**Table 3:** *RC* circuit fitting parameters (error < 5%) of complex impedance semicircles at different temperatures (280 K–140 K).

Temp. (K)	R_g_ (MΩ)	**CPE**_**g**_	n_g_	C_g_	R_gb_ (MΩ)	CPE_gb_	n_gb_	C_gb_	C_surface layer_
280	0.47	6.25 × 10^−12^	0.93	2.96 × 10^−12^	0.47	1.89 × 10^−11^	0.88	5.86 × 10^−12^	3.21 × 10^−7^
260	19.0	1.54 × 10^−12^	0.92	3.52 × 10^−12^	9.88	7.71 × 10^−12^	0.94	4.42 × 10^−12^	1.30 × 10^−8^
240	38.8	5.93 × 10^−12^	0.95	2.99 × 10^−12^	21.9	5.04 × 10^−12^	0.96	3.48 × 10^−12^	2.01 × 10^−9^
220	65.6	3.19 × 10^−11^	0.92	1.61 × 10^−11^	24.1	7.04 × 10^−12^	0.98	5.70 × 10^−12^	1.97 × 10^−9^
200	14.1	2.93 × 10^−11^	0.91	1.08 × 10^−11^	36.7	9.47 × 10^−12^	0.98	7.00 × 10^−12^	1.53 × 10^−9^
180	10.9	4.16 × 10^−11^	0.89	1.20 × 10^−11^	57.9	7.57 × 10^−12^	0.97	5.95 × 10^−12^	1.19 × 10^−9^
160	1.30	5.16 × 10^−11^	0.91	1.64 × 10^−11^	65.0	8.86 × 10^−12^	0.98	6.48 × 10^−12^	2.50 × 10^−9^
140	3.50	7.70 × 10^−11^	0.93	2.69 × 10^−11^	77.0	6.60 × 10^−12^	0.97	6.77 × 10^−12^	4.10 × 10^−9^

**Figure 6:**
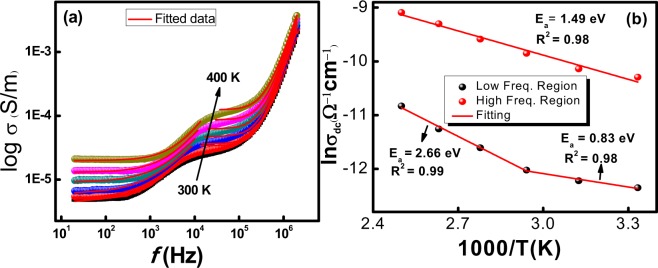
(**a**) Effect of frequency and temperature on ac conductivity, (**b**) Arrhenius curve between dc parts of conductivity with temperature.

The entire ac conductivity (σ‘(ω)) of BiSiO nanofibers recorded in temperature range of 300 K–400 K and frequency range of 20 Hz to 2 MHz is represented in Fig. 6(a). The ac conductivity of BiSiO nanofibers is described by three regions; (*i*) two plateau regions and (*ii*) one dispersion region. The second plateau region appeared at intermediate frequencies. The size of the second plateau is decreased with an increase in temperature. It is very common in heterogeneous nanofibers. This additional plateau is attributed to difference in conductivity of bismuth and silicate network. This type of conduction mechanism is also represented for former heterogeneous nanofibers and bulk counterparts^27^. The increase in electrical conductivity of BiSiO nanofibers with temperature might be due to increase in drift mobility of thermally triggered charge carriers^28,29^.

Generally, the ac conductivity of hopping charges for various semiconducting materials obeys Jonsher’s power law^29^.

5 $$\sigma {\prime} ={\sigma }_{0}+A{\omega }^{s}$$

Where *σ*_0_ is *dc* conductivity, *A* is the pre-exponential factor and *s* is the frequency exponent (0–2)^30^. The values of dc conductivity and *s* are presented in tabular form (Table 4). Above mentioned Eq. 5 can not only explains the experimental data of *ac* conductivity for BiSiO nanofibers but to some extent can describe the existence of two relaxation mechanisms. Therefore, each *ac* conductivity curve is divided into two segments. Then results of each part is autonomously fitted with Jonsher’s power law. Each region shows good fitting with Eq. 5. For low and high frequencies plateaus, the observed values of *σ*_0_ and *s* are acquired individually. The presence of second plateau in the midway to high frequency region in BiSiO nanofibers demonstrated heterogeneous structure with different phases, indicating the Maxwell-Wagner effect^17^; which is usually detected in the mixt systems wherever the difference in conductivity values for two parts is prominent^17,27^.

**Table 4:** Jonsher’s power law fitting parameters of ac conductivity at different temperatures.

Temp. (K)	Low frequency region				High frequency region			
	σ_o_	A	s	R^2^	σ_o_	A	s	R^2^
300	4.33 × 10^−6^	2.33 × 10^−8^	0.757	0.98	3.38 × 10^−5^	1.39 × 10^−16^	2.09	0.99
320	4.93 × 10^−6^	1.61 × 10^−8^	0.809	0.99	3.63 × 10^−5^	1.49 × 10^−16^	2.09	0.98
340	6.01 × 10^−6^	2.07 × 10^−8^	0.793	0.98	4.72 × 10^−5^	1.95 × 10^−16^	2.08	0.97
360	9.10 × 10^−6^	1.09 × 10^−8^	0.875	0.97	6.23 × 10^−5^	2.63 × 10^−16^	2.07	0.99
380	1.29 × 10^−6^	509 × 10^−8^	0.955	0.99	8.06 × 10^−5^	2.96 × 10^−16^	2.06	0.98
400	1.98 × 10^−6^	1.09 × 10^−8^	1.097	0.98	1.24 × 10^−4^	4.21 × 10^−16^	2.05	0.99

Conduction mechanism in BiSiO nanofibers is further analyzed by investigating the deviation of frequency exponent (*s*) with increase in temperature and frequency. These two variations explained either the conduction mechanism due to classical hoping of charge carrier above barrier or the quantum mechanical tunneling (QMT) or overlapping the large polaron tunneling^27^. However, in presently studied heterogeneous nanofibers, no frequency dependent of *s* is observed in the chosen frequency range. Therefore, the overall conduction mechanism is based on the temperature dependent *s* component. In this work, for low frequency regions where *s* < 1 the hopping involves a translational motion with an unexpected hopping; however for high frequency region where *s* > 1, the motion implicates localized hopping deprived of the species leaving the locality^28^. The variation of *s* as a function of temperature predicts the origin of conduction mechanism. For low frequency region the value of *s* increases with increase in temperature which can be explained by the large polaron QMT model^19^.

The annealing at 600 °C of BiSiO nanofibers causes the creation of bismuth vacancies inevitably due to volatility of Bi. In order to maintain charge neutrality; oxygen vacancies are produced at the same time. It is reported that the ionization of oxygen vacancies either singly or doubly ionized create weakly bonded electrons which take part in conduction at relatively higher temperature than room temperature^31^. Singly ionized oxygen vacancies have activation energy in the range of 0.3–0.7 eV while doubly ionized oxygen vacancies need activation energy of 0.7–1.2 eV^32^.

Arrhenius relation is used here to calculate the *E*_*a*_ from the well fitted data of two regions. The correlation coefficient value is very close to unity stating the goodness of fitting.

6 $${\sigma }_{o}={\sigma }_{a}\exp (-\frac{{E}_{a}}{{K}_{B}T})$$

Where *σ*_*o*_ is the dc conductivity, *σ*_*a*_ is the pre-exponential factor, *k*_*B*_ is the Boltzmann constant and *Ea* is the activation energy. It is worth noting that ln σ_o_ vs. 1000/T can be fitted in two linear relations for region 1 with refined variation in slope at two temperatures ranges i.e. (1) T < 360 K and (2) T > 360 K that give the values of *E*_*a*_ ~ 2.66 eV and 0.83 eV correspondingly as shown in Fig. 6(b). For T < 360 K, the *E*_*a*_ = 2.66 eV indicates the restriction in generation of the oxygen vacancies and formation of other types of defects such as space charge electrons. It can be supported by the interpretation of *IV* curves in the next section. While the *E*_*a*_ = 0.83 eV shows short range hopping motion of doubly ionized oxygen vacancies. For region 2, the *E*_*a*_ value is 1.49 eV. This phenomenon is also observed in ZrTiO_3_ as reported by Zhang *et al*.^32^. Furthermore, the *E*_*a*_ values estimated from conductivity are very close, which recommends that the electrical conductivity and the relaxation process may be endorsed by sort of similar charge carriers^18^.

**Figure 7:**
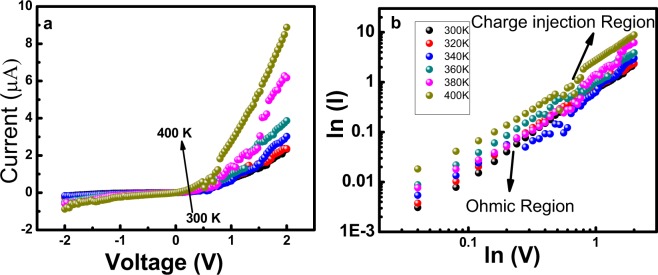
(**a**) IV characteristics of bismuth silicate nanofibers at different temperatures (**b**) IV curve on *ln-ln* scale.

Current- Voltage (*IV*) characteristics were performed in the temperature range of 300 K–400 K for BiSiO nanofibers. Figure 7(a) shows the temperature dependent *IV* curves showing semiconducting behavior (Nonlinear). The typical *IV* characteristics show diode-like behavior^33^. Different factors can affect the *IV* characteristics *e.g*. device fabrication, surface density, nature of contact, temperature, applied field, nanostructure type and material etc. Different types of contact and bulk limited conduction mechanisms can occur in device. The rectifying diode-like behavior^34^ can be further investigated using *ln-ln* plot. In order to investigate the possible conduction mechanism, we have plotted the *IV* curves on *ln-ln* scale as shown in Fig. 7b. The curves show two distinct regions having different slope values. The prominent region at lower voltages having slope ~1 is known as ohmic region^33^. This region shows that our nanofibers and electrical contact material have negligible work function difference. However, at higher voltages the slope of *IV* curves changes and charge injection region can be seen which may be followed by space charge limited conduction at higher voltages. This type of conduction can be seen in our previous research reports published elsewhere^14^. The increase in slope and absence of ohmic region indicates the charge injection region (interface between material and electrical contact). The charge injection region occurs due to difference of injected carriers and carriers present inside material (BiSiO nanofibers) which may lead to the space charge limited current conduction.

Both *IV* curves and impedance spectroscopy results are in agreement with each other showing the possibility of space charge region. Space charge region or interface region in both cases actually influences the properties of material. Different type of interfaces can play a vital role in our conduction mechanism e.g. interface region between nanofibers, interface between nanofibers and contact region and interface of nanofibers with the environment^35^.

## Conclusion

In summary, the BiSiO nanofibers were synthesized using electrospinning technique followed by heat treatment at 600 °C in an air furnace. The structural, complex impedance, conductivity and *dc* properties were investigated thoroughly. The *ac* study revealed the formation of two relaxation phenomenon in semiconducting BiSiO nanofibers both at (300–400 K) and (280 K–140 K). Moreover, the conduction mechanism was elucidated by large polaron QMT model followed by Maxwell-Wagner effect. The overall analysis of electrical measurements showed the NTCR character of the nanofibers. The *IV* characteristics exhibited space charge limited current conduction at intermediate voltage which was in agreement with impedance data.
